# Around the Hospitals

**Published:** 1895-08-17

**Authors:** 


### Around the Hospitals.

[Notice to the Managers of Institutions.—Special space is now reserved for the insertion of notices of hospital meetings and festivals. The Editor requests that all notices may reach The Hospital Office, 428, Strand, at least one week before the date of each meeting.]

**Leeds Hospital for Women and Children.**—This hospital has witnessed several changes and additions during the past year. Structural improvements amounting to £1,000 have been effected; a house surgeon has been appointed, and the office of matron in distinction to head nurse has been created, and also that of sister in place of nurse. The hospital has benefited much from that excellent association in Leeds, the Ladies' Hospital Fund. The work-people of Leeds also set a good example to other communities by their generosity towards the medical charities. In respect to annual subscriptions the hospital has not fared so well, and a decrease is visible. This is to be regretted, as the work of the institution is likely to increase, especially in the out-patients' department. Here the numbers of the past year have exceeded those of 1893 by 79. The in-patients in 1894 numbered 269 and the out-patients 2,306. The total income amounted to £2,595, and the expenditure to £2,190. Under the rule which provides for the investment of legacies, a deficiency of £7 10s. is shown on the accounts.

